# A novel peptide RIFV suppresses human adipocyte differentiation through the inhibition of C/EBP-β expression

**DOI:** 10.1186/s12986-019-0414-z

**Published:** 2019-12-23

**Authors:** Wen Zhang, Dan Shen, Yun Li, Hong Zhong, Xing Wang, Xian-wei Cui, Chun-Mei Shi, Chen-Bo Ji, Xi-Rong Guo, Ling Chen

**Affiliations:** 1 Department of Pediatrics, Nanjing Maternity and Child Health Care Hospital, Women’s Hospital of Nanjing Medical University, Nanjing, 210029 China; 2 Department of Internal Medicine, Nantong Maternal and Child Health Care Hospital, Affiliated to Nantong University, Nantong, China; 3 Hongqiao International Institute of Medicine, Tongren Hospital, Shanghai Jiao Tong University School of Medicine, Shanghai, China

**Keywords:** RIFV, Peptides, Adipocyte differentiation, C/EBP-β

## Abstract

**Background:**

Obesity is a global epidemic disease that increases the risk of metabolic syndrome. However, therapeutic drugs for obesity are still scarce. In recent years, peptides have been identified as new biological regulators. RIFV (R-I-F-V-P-I-K-G-R-P-A-P), a novel active peptide from our peptide database.

**Methods:**

We performed oil red O staining and triglyceride measurement to analyze the influence of RIFV on white preadipocytes differentiation. Then the effects of RIFV on cell proliferation, apoptosis and cell cycle were determined by using CCK-8 assay and flow cytometry. The mRNA and protein levels of adipogenesis-related genes were respectively detected by qRT-PCR and western blot. Rescue experiment was conducted to confirm whether RIFV could regulate adipocytes differentiation via targeting C/EBP-β. Finally, the luciferase reporter gene assay was performed to verify the regulation of RIFV on C/EBP-β gene.

**Results:**

RIFV was revealed to inhibit the differentiation of human white adipocytes without affecting their proliferation. Additionally, RIFV could also suppress the differentiation of mouse primary white preadipocytes isolated from inguinal fat tissues. Furthermore, RIFV may have an inhibitory effect on adipogenesis by inhibiting the regulation of the adipogenic gene C/EBP-β.

**Conclusions:**

Our results indicated that RIFV may be a novel essential regulator of adipocyte differentiation and represents a therapeutic strategy for obesity and related complications.

## Introduction

Obesity is ascribed to excessive fat accumulation, which is a major risk factor for many metabolic diseases, such as hypertension, diabetes, cardiovascular disease and certain types of cancer [1, 2]. Obesity has been a worldwide public health problem. However, therapeutic medicine to treat obesity is still limited. Most of clinical medications play a role in weight loss mainly via inhibiting appetite, which is often associated with side effects [3, 4]. Therefore, it is very important to find new therapies to treat obesity.

Previous studies have suggested that various substances, such as microRNA [5, 6], animal venom [7] and plant extracts [8], could affect the formation and metabolism of adipocytes by affecting the functions of important transcription factors. Lot of researches had focused on the therapeutic potential of these substances in the treatment of obesity. However, animal venom or plant extracts are usually a complex mixture and are more likely to have side effects [7, 8]. In comparison, microRNA is with single component and functions mostly by targeting specific mRNA gene [5, 6]. It’s worth noting that some microRNAs based drugs have been utilized in clinical trials to treat different disease. However, there is few about obesity [9, 10].

In recent years, as a new biological regulator, peptides have attracted increasing attention, which have been involved in many physiological and pathological processes, such as cardiovascular disease [11], infection [12], diabetes [13] and obesity [14]. Until now, some antiobesity peptides have already been utilized as therapeutic agents in clinical application. For instance, the analog of glucagon-like peptide 1 (GLP-1), liraglutide, has shown greater weight loss by decreased food intake and delayed gastrointestinal emptying [15]. Beyond liraglutide, the US FDA has already approved a total of six GLP-1-based drugs: exenatide, lixisenatide, liraglutide, semaglutide, dulaglutide and albiglutide [16]. In the clinic, doctors can prescribe these medications to patients with obesity or type 2diabetes by extending the duration of action on the GLP-1 receptor. Notably, GLP-1 and its analogs also have unwanted side effects, such as nausea and pancreatitis [17, 18].

Recently, researchers have paid attention to some alternative peptides related to obesity treatment owing to their potential for animals without causing significant adverse effects. For instance, the analog of the sea anemone-derived peptide ShK, ShK-186, has been implicated in lowering the body weight gain, lipid accumulation, and inflammatory infiltration of high fat-induced obese mice [19, 20]. The polypeptide pNaKtide, which is from the nucleotide-binding domain of the α subunit of Na/K-ATPase, can attenuate lipid accumulation and oxidative stress in 3T3L1 adipocytes in a dose-dependent manner by antagonizing the Na/K-ATPase–mediated amplification of ROS signaling. In vivo, the treatment of obese mice with pNaKtide showed reduced bodyweight gain and improved insulin sensitivity [21].

In this study, we screened a novel peptide, termed RIFV (sequence R-I-F-V-P-I-K-G-R-P-A-P), which contains 12 amino acids. RIFV was identified from our peptide database, which was established from human tissues, blood and other body fluids. Through functional screening, we identified that RIFV could inhibit human adipocyte differentiation but did not influence proliferation and apoptosis of these cells. In addition, we found that the inhibition effect of RIFV on differentiation was conserved in mouse primary white preadipocytes isolated from inguinal fat tissues. Furthermore, RIFV may have an inhibitory effect on adipogenesis by inhibiting the regulation of the adipogenic gene C/EBP-β. Our results indicate that RIFV acts as a negative regulator of adipogenesis.

## Materials and methods

### Cell culture and differentiation of human white preadipocytes

Human white preadipocytes (hWA; purchased from ScienCell Research Laboratories) were cultured and maintained in preadipocyte medium (PAM; ScienCell Research Laboratories) containing 5% fetal bovine serum (FBS), 1% preadipocyte growth supplement(PAGS), and 1% penicillin/streptomycin(P/S) at 37 °C in a humidified atmosphere under 5% CO_2_. After 2 days of cell culture, the confluent cells were exposed to differentiation medium for 4 days. The induction medium contained PAM without serum, supplemented with 0.5 mM 3-isobutyl-1-methylxanthine (IBMX; Sigma, St. Louis, MO, USA), 100 nM insulin (Sigma), 1 μM dexamethasone (Sigma), and 1 μM rosiglitazone (Sigma). Then, the medium was changed to serum-free PAM with 10 nM insulin every 2 days until the accumulation of lipid droplets was observed.

### Isolation and differentiation of mouse white preadipocytes

Mouse primary white preadipocytes (mWA) were isolated from the stromal vascular fraction (SVF) of the inguinal WAT of 4- to 6-week-old male C57BL/6 J mice (purchased from Model Animal Research Center of Nanjing University, China). All animal studies were approved by the Ethics Committees at the Nan Jing Medical University. The detached tissues were cut into small pieces in Dulbecco’s modified Eagle’s medium (DMEM; Gibco, Carlsbad, CA) containing 5% FBS (Gibco) and 0.2% collagenase I (Sigma-Aldrich, St. Louis, MO). After 60 mins of digestion at 37 °C, the digestion buffer was filtered through a 100 μm nylon mesh (Thermo Fisher Scientific, Waltham, MA, USA) and centrifuged at 1800 rpm for 10 mins. The pellet was then resuspended in DMEM growth medium (GM), supplemented with 10% FBS (Gibco) and 1% P/S (Gibco), and seeded onto a 6-well plate.

After two more days of 100% cell confluence, the culture medium was replaced by preadipocyte differentiation medium (PADM; ScienCell), containing 5% FBS (ScienCell), 1% preadipocyte differentiation supplement (PAdDS; ScienCell) and 1% P/S (ScienCell). Seven days after differentiation, the medium was changed to GM as previously described. The cells were then maintained in GM for 7 days when the lipid droplets could be sufficiently displayed. During the whole process of culture and differentiation, the medium was changed every 2 days.

### Oil red O staining

Mature adipocytes were washed twice with phosphate-buffered saline (PBS; ScienCell) and then fixed in 4% paraformaldehyde for 30 mins. After washing with PBS, the cells were incubated with 0.2% Oil Red O (Sigma) solution for 30 mins at 37 °C. Then, the stained cells were washed with PBS and visualized under a microscope (CarlZeiss, Werk Gottingen, Germany).

### Triglyceride measurement

After washing twice with PBS, the intracellular triglycerides of mature adipocytes were determined by using a tissue triglyceride assay kit (Applygen Technologies Inc., Beijing, China). According to the manufacturer’s instructions, the mature adipocytes were harvested in lysis buffer, and the cell lysates were homogenized by centrifugation and reacted with reagents. The absorbance values were measured at a wavelength of 550 nM. Subsequently, the protein content was determined by using the BCA Protein Assay Kit (Thermo Fisher Scientific, Waltham, MA). Finally, the triglyceride content was normalized to the total protein content.

### Cell proliferation assay

For the cell proliferation assay, a WST-8 Cell Counting Kit-8 was used (CCK-8; Dojindo Molecular Technologies, Japan) as reported previously [22]. Human white preadipocytes (1000 cells/well) were seeded onto a 96-well plate in PAM containing 5% FBS, 1% PAGS and 1% P/S and stimulated with RIFV (0 μM, 10 μM, 50 μM, and 100 μM) every day. The cells were analyzed at 0 h, 24 h, 48 h and 72 h. Then, the old medium was replaced with fresh PAM supplemented with 10% CCK-8 reagent. After incubation at 37 °C for 60 min, the absorbance was measured by using a Multiskan MK3 microplate reader (Thermo Fisher Scientific) at 450 nm. Finally, the proliferation curve was calculated by using these values.

### Cell apoptosis assay

The human white preadipocytes were cultured on a 6-well plate with PAM. After the cells were cultured to 80% confluence, they were stimulated with RIFV (0 μM, 10 μM, 50 μM, and 100 μM) every day. After 2 days, the cells were collected, washed twice with PBS and stained with the Annexin V-FITC/PI Apoptosis Detection Kit (Beyotime Institute of Biotechnology, China) according to the manufacturer’s instructions. The cell apoptosis distribution was analyzed using flow cytometry. The fraction of the cell population in different quadrants was analyzed by using quadrant statistics. Cells in the lower left quadrant represent survival, the lower right quadrant represents apoptosis and the upper right quadrant represents necrosis or post-apoptotic necrosis.

### Cell cycle assay

The human white preadipocytes were seeded and cultured on a 6-well plate. After the cell density reached 80%, the preadipocytes were treated with different concentrations of RIFV (0 μM, 10 μM, 50 μM, and 100 μM). After incubation for 48 h, the cells were serum-deprived for 12 h for cell cycle synchronization and then incubated in PAM containing 5% FBS for another 36 h.

Then, the cells were collected by using trypsin/EDTA (Sigma) and centrifuged at 1500 rpm. The cell pellets were washed twice with PBS and fixed in 75% precooling ethanol (Sigma). After staining with the Cell Cycle and Apoptosis Analysis Kit (Beyotime) according to the manufacturer’s instructions, the cell cycle distribution was analyzed by using flow cytometry.

### Protein extraction and western blot analysis

Mature adipocytes were harvested in cold RIPA lysis buffer (Beyotime, China) containing 1% phenylmethylsulfonyl fluoride (PMSF, Beyotime).The concentrations of protein were determined by using a BCA protein assay kit (Thermo Fisher Scientific). Equal amounts of protein were loaded and analyzed by10% SDS-PAGE and transferred to 0.2-μm PVDF membranes. After blocking with 5% nonfat dry milk, the membranes were probed with the following primary antibodies overnight at 4 °C: rabbit polyclonal β-actin (Biosharp, China, BL005B, diluted 1:1000), monoclonal rabbit PPAR-γ (Abcam, ab178860, diluted 1:1000), polyclonal rabbit C/EBP-β (Abcam, ab32358, diluted 1:1000), and C/EBP-α (Abcam, ab40761, diluted 1:1000). The membranes were washed 3 times with TBST and incubated with peroxidase-conjugated goat anti-rabbit IgG (Biosharp, China, BL003A, diluted 1:5000) for 1 h at room temperature. All bands were visualized by using Beyo ECL Plus (Beyotime, China, P0018).

### RNA extraction and real-time quantitative PCR (qRT-PCR)

Total RNA was isolated from adipocytes by using Trizol reagent (Invitrogen, CA, USA) and extracted by using the RNA prep-pure Cell/Bacteria Kit (TianGen, Beijing, China). cDNA was synthesized from 1 μg of total RNA by using the Prime Script™ RT reagent Kit with g-DNA Eraser (TAKARA BIO, Beijing, China). Real-time PCR was performed with SYBR-Green (Roche, Life Technologies). Measurements were calculated by the 2-^△△Ct^ method and normalized to PPIA. All primers used are listed in Table 1. All real-time PCR reactions were run on a V7 Fast Real-Time PCR system (Applied Biosystems).

**Table 1:** Primers sequence used in the paper

	Direction	Sequence(5′-3′)
hC/EBP-α	Forward	TGGACAAGAACAGCAACGAG
	Reverse	TTGTCACTGGTCAGCTCCAG
hC/EBP-β	Forward	GACAAGCACAGCGACGAGTA
	Reverse	AGCTGCTCCACCTTCTTCTG
hPPAR-γ	Forward	GCTGTGCAGGAGATCACAGA
	Reverse	GGGCTCCATAAAGTCACCAA
mC/EBP-α	Forward	TGCGCAAGAGCCGAGATAAA
	Reverse	CCTTCTGTTGCGTCTCCACG
mC/EBP-β	Forward	AGCGGCTGCAGAAGAAGGT
	Reverse	GGCAGCTGCTTGAACAAGTTC
mPPAR-γ	Forward	GTGCCAGTTTCGATCCGTAGA
	Reverse	GGCCAGCATCGTGTAGATGA
mATGL	Forward	TTCACCATCCGCTTGTTGGAG
	Reverse	AGATGGTCACCCAATTTCCTC
mFABP4	Forward	AAGGTGAAGAGCATCATAACCCT
	Reverse	TCACGCCTTTCATAACACATTCC

### DNA plasmid construction and transduction

The C/EBP-β overexpressing vector (pcDNA3.0-C/EBP-β, Genecreate, Wuhan, China) was synthesized by inserting the whole open reading frame of human C/EBP-β into pcDNA3.0. According to the manufacturer’s instructions of.

Lipofectamine3000 (Invitrogen), 2.5 μg C/EBP-β plasmids or negative control plasmids were separately mixed with 5ul Lipo3000 transfection reagent and then added to hWA cells cultured in 6-well plates.To maximize the overexpression efficiency, hWA cells were transfected with plasmids for 2 days before differentiation and repeated at Day 4 of differentiation. Then, the cells were collected at Day 4 and Day 8 of differentiation, and various experiments were carried out subsequently.

### C/EBP-β promoter luciferase reporter assay

The full-length of human C/EBP-β promoter was synthesized by PCR and inserted downstream of the luciferase gene in the pGL3-Basic firefly luciferase reporter. In the luciferase assays, the C/EBP-β promoter luciferase plasmid was transiently transfected into hWA cells cultured in 24-well plates using Lipofectamine2000 (Invitrogen) according to the manufacturer’s instructions. Subsequently, the hWA cells were treated with 50 μM RIFV. After 72–96 h, cell lysates were analyzed for luciferase activity using the Dual-luciferase Reporter Assay System (Promega Corporation).

### Statistical analysis

The data were analyzed by using SPSS 17.0. All values are presented as the means ± SEM of at least three independent experiments. Significance of differences between two groups was analyzed by Student’s t test. For comparing more than two means, one-way analysis of variance (ANOVA) was employed. *P* < 0.05 was considered to be statistically significant.

## Results

### RIFV can enter human white preadipocytes and inhibit cell differentiation

**Fig. 1:**
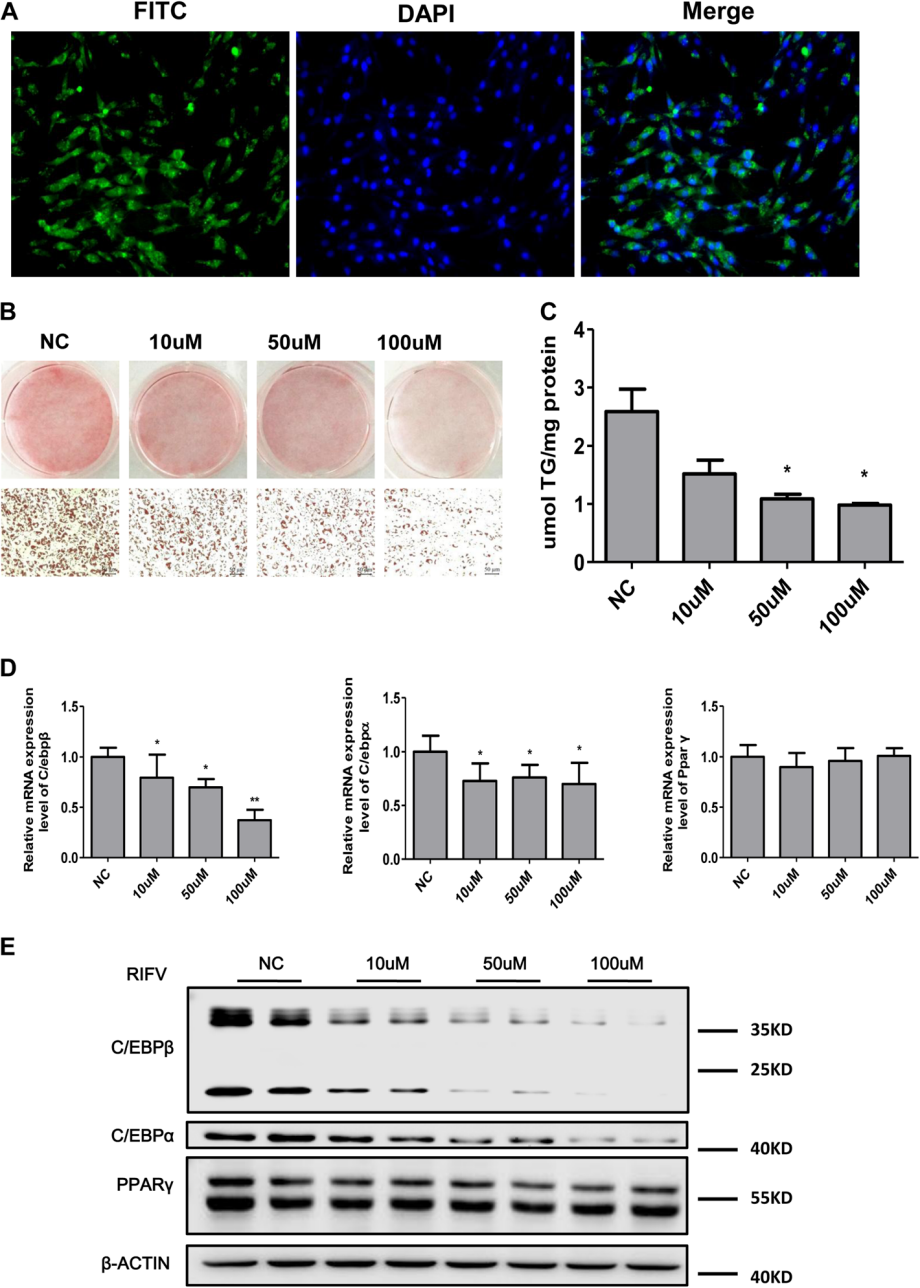
Effects of RIFV on the differentiation of human white adipocytes. **a** FITC-labeled RIFV was added to the culture medium of human preadipocytes for 48 h, and RIFV could successfully enter the human preadipocytes. **b** Human preadipocytes were cultured with RIFV (NC, 10 μM, 50 μM and 100 μM) and differentiated for 8 days to mature adipocytes. Then, the cells were stained with Oil Red O and visualized under a light microscope (× 200). **c** The cellular triglyceride contents of human mature adipocytes on differentiation day 8 were measured using a commercial TG assay kit. **d** The mRNA expression levels of the indicated genes C/EBP-β, C/EBP-α and PPAR-γ in mature human adipocytes treated with RIFV on day 4. Each value was normalized to PPIA. **e** The protein levels of the indicated genes C/EBP-β, C/EBP-α and PPAR-γ of mature human adipocytes treated with RIFV at day 4 of differentiation. Each value was normalized to β-actin. The data are expressed as the means ± SD, *n* = 3. *P < 0.05, **P < 0.01.Abbreviations: NC, negative control; C/EBP-β, CCAAT/enhancer binding protein beta; C/EBP-α, CCAAT/enhancer binding protein alpha; PPAR-γ, peroxisome proliferator-activated receptor gamma

First, we utilized FITC to label peptide RIFV and found that FITC-conjugated RIFV could successfully enter human preadipocytes, as shown in Fig. 1a. To examine the role of RIFV on the differentiation of hWA, we added several different concentrations of RIFV (0 μM, 10 μM, 50 μM and 100 μM) to hWA during their differentiation. Then, Oil Red O staining and TG measurement were used to determine the effect of RIFV on intracellular lipid accumulation in mature adipocytes at day 8 of differentiation. As shown in Fig. 1b and c, RIFV treatment could lead to significantly reduced lipid accumulation, and the inhibitory effect of RIFV gradually increased with increasing RIFV concentration.

Several transcription regulators, such as CCAAT/enhancer-binding protein beta (C/EBP-β), CCAAT/enhancer-binding protein alpha (C/EBP-α) and peroxisome proliferator-activated receptor gamma (PPAR-γ), were reported to mediate adipogenesis. To investigate the mechanism by which RIFV inhibited human white preadipocyte differentiation, we measured the mRNA expression of these markers at day 4 by qRT-PCR. The results showed that the expression of C/EBP-β and C/EBP-α, but not PPAR-γ, at the mRNA level was downregulated in response to RIFV treatment (Fig. 1d). In addition, western blotting was carried out, and the same result was observed in Fig. 1e. Thus, our data demonstrated that RIFV could inhibit human adipocyte differentiation.

### RIFV does not influence the proliferation, apoptosis or cell cycle of hWA

**Fig. 2:**
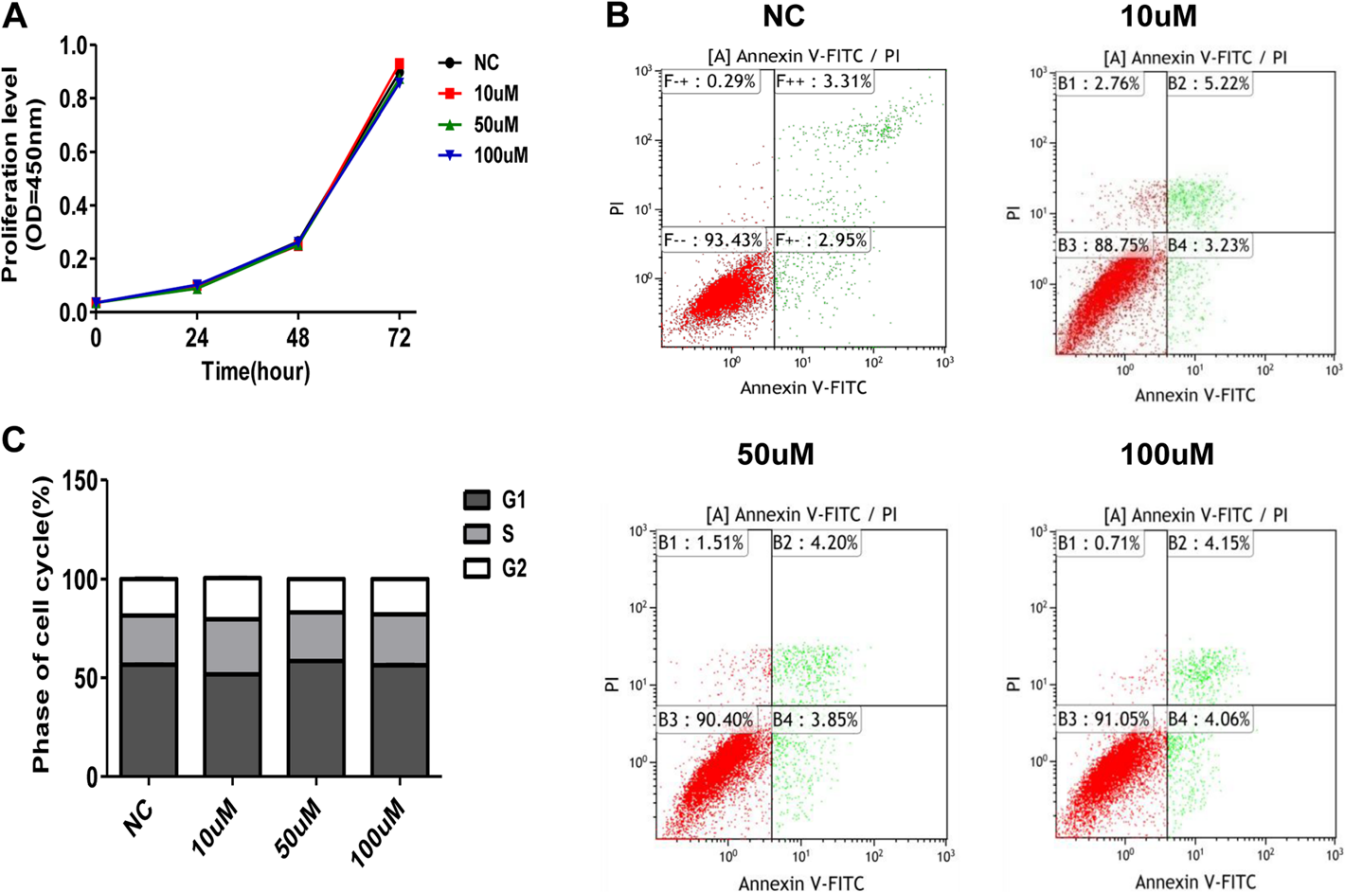
Peptide RIFV did not influence the proliferation, apoptosis or cell cycle of human white preadipocytes. **a** Cells were cultured in PAM containing RIFV (NC, 10 μM, 50 μM and 100 μM) for 24 h, 48 h, 72 h, and the cell proliferation level was assessed via a CCK-8 assay. **b** Apoptosis and **c** cell cycle of human white preadipocytes treated with RIFV for 24 h were analyzed by flow cytometry. The values represent the means ± SD, *n* = 3. *P < 0.05, **P < 0.01

A CCK-8 assay was used to assess whether RIFV had the ability to affect the proliferation of hWA. Cell viability was detected at 24 h, 48 h, and 72 h after treatment with RIFV (0 μM, 10 μM, 50 μM and 100 μM). The results showed that RIFV stimulation did not influence the proliferation of hWA (Fig. 2a). Additionally, we observed that the apoptosis and cell cycle of hWA after RIFV treatment were also comparable to that of the negative control (Fig. 2b and c).

### RIFV also inhibits the differentiation of mouse primary white preadipocytes

**Fig. 3:**
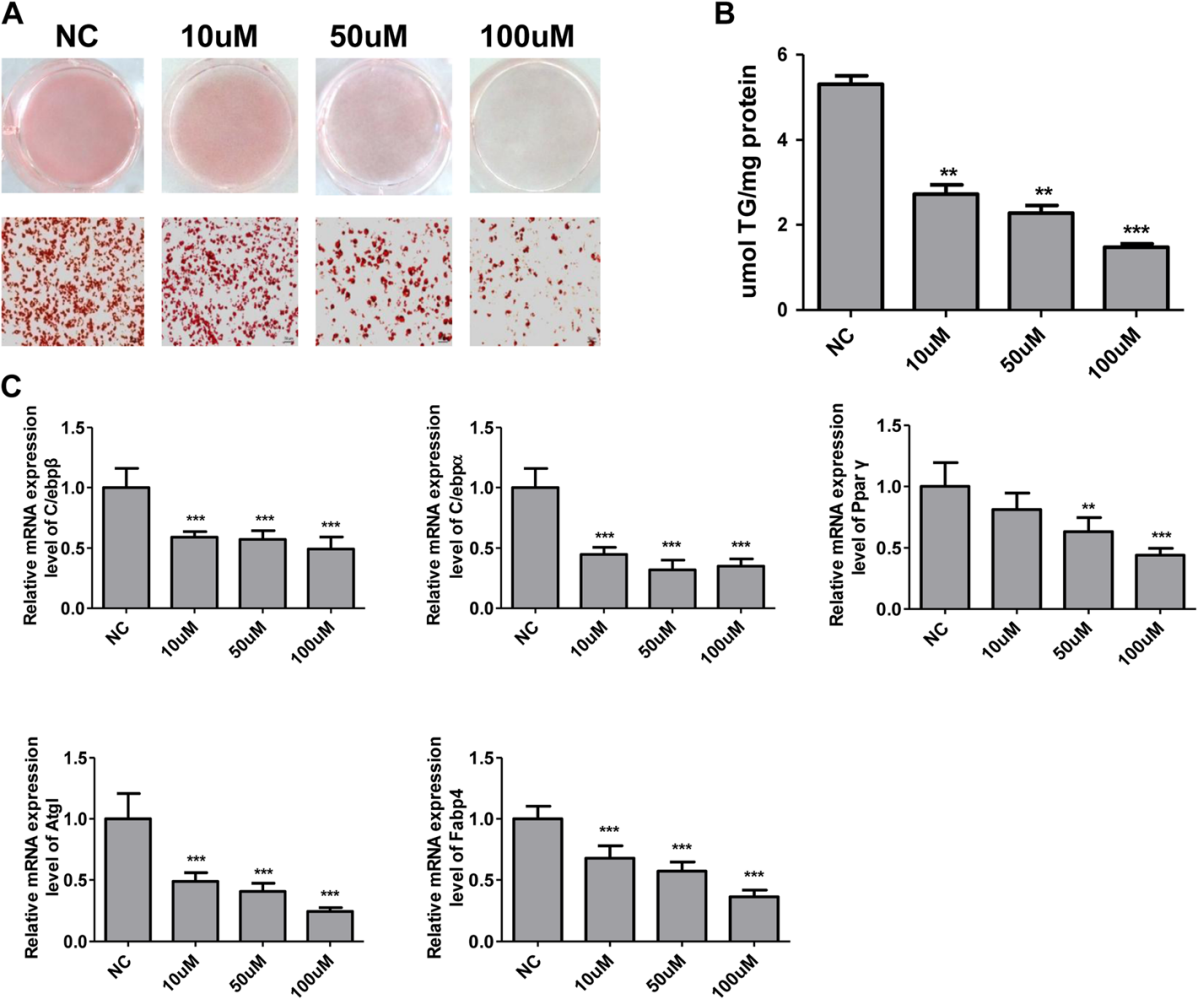
Effects of RIFV on the differentiation of mouse primary white adipocytes. **a** The mouse primary white preadipocytes were isolated and cultured with RIFV (NC, 10 μM, 50 μM and 100 μM) and then differentiated into mature adipocytes. The cells were stained with Oil Red O and visualized under a light microscope (× 200). **b** The cellular triglyceride contents of mouse mature adipocytes were measured by a commercial TG assay. **c** The mRNA expression levels of the indicated genes C/EBP-β, C/EBP-α, PPAR-γ, ATGL and FABP4 in mature mouse adipocytes treated with RIFV at day 4 of differentiation. Each value was normalized to PPIA. The data are expressed as the means ± SD of three independent experiments. *P < 0.05, **P < 0.01.Abbreviations: ATGL, adipose triglyceride lipase; FABP4, fatty acid binding protein 4

As we revealed that RIFV could inhibit hWA differentiation, we then investigated whether RIFV had the same inhibitory effect on mouse primary white preadipocyte differentiation. The mWA cells were separately incubated with 10 μM, 50 μM and 100 μMRIFV during differentiation. Compared to the negative control, the intervention of RIFV resulted in the suppression of adipocyte differentiation, as determined by oil red O staining on day 8 of differentiation (Fig. 3a). This result is consistent with the decrease observed in TG production (Fig. 3b).In addition, we detected the mRNA expression levels of adipogenic transcription factors and adipocyte-specific genes, including C/EBP-β, C/EBP-α, PPAR-γ, ATGL (adipose triglyceride lipase) and FABP4 (fatty acid binding protein 4), on the indicated days after the induction of differentiation. RIFV significantly reduced the expression of the transcription factors C/EBP-β, C/EBP-α and PPARγ on day 4 (Fig. 3c). Moreover, the expression levels of ATGL and FABP4 were also downregulated on day 8.

### The hWA differentiation was inhibited by RIFV only at the early stage

**Fig. 4:**
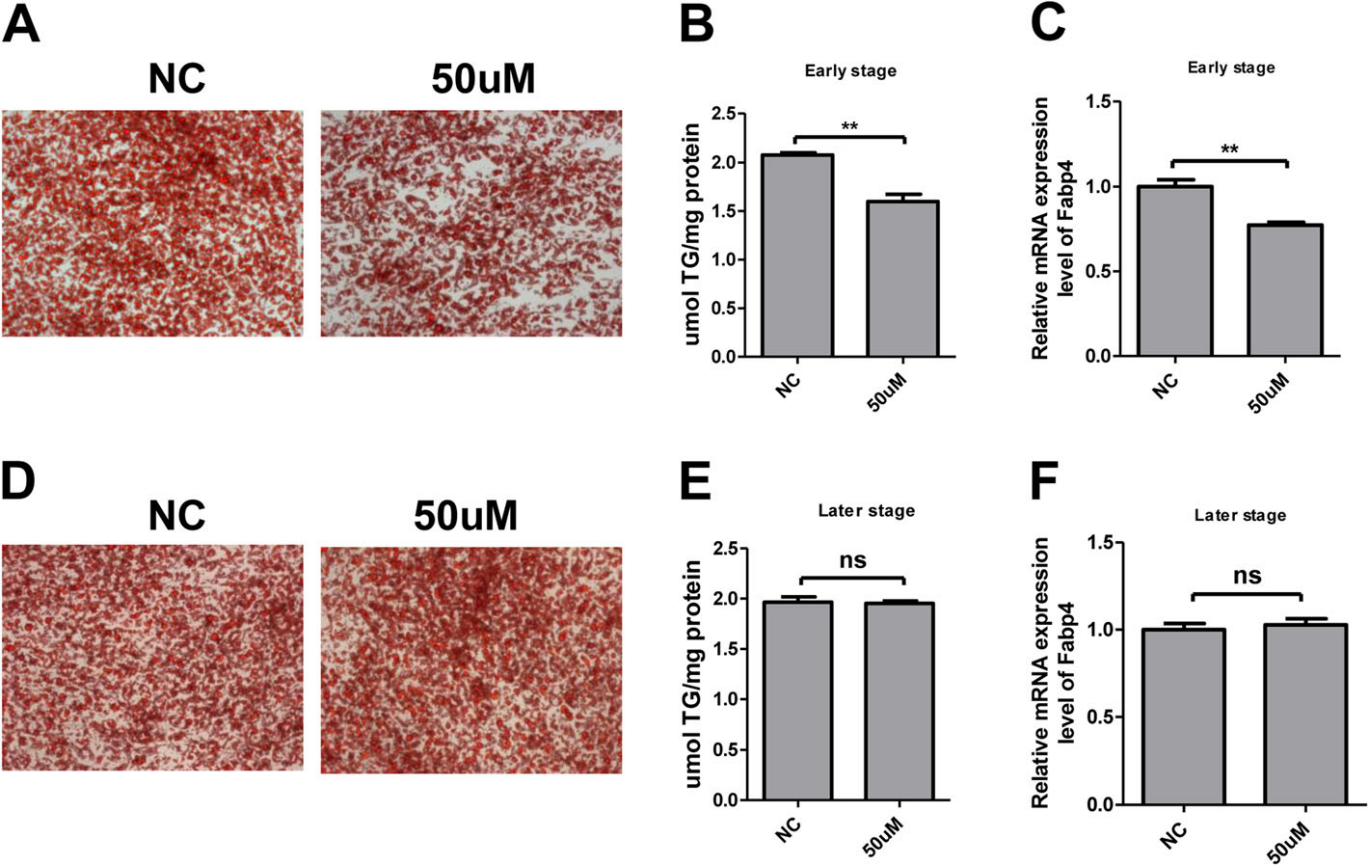
The human white adipocytes differentiation was inhibited by RIFV at the early stage. **a**–**c** The human preadipocytes were cultured with 50 μM RIFV from Day0 to Day3 during adipogenesis. Then the mature adipocytes were stained with Oil Red O and visualized under a light microscope (× 200) on differentiation Day8 **a**. The cellular triglyceride contents of mature adipocytes were measured **b**. The mRNA expression levels of FABP4 were analyzed by q-RT-PCR **c**. **d**–**f** The human adipocytes were cultured with 50 μM RIFV from Day6 to Day8 during adipogenesis. Then the mature adipocytes were stained with Oil Red O and visualized under a light microscope (× 200) on differentiation Day8 **d**. The cellular triglyceride contents of mature adipocytes were measured by a commercial TG assay **e**. The mRNA expression levels of FABP4 were analyzed by q-RT-PCR **f**. The data are expressed as the means ± SD of three independent experiments. **P < 0.01.ns. no significance

The above results showed that C/EBP-β was significantly decreased at both the mRNA and protein levels in hWA cells after RIFV treatment. Considering that C/EBP-β is an early adipocytes differentiation associated transcription factor, we treated human adipocytes with RIFV separately in the early or later stage of adipogenesis. At the early stage, we added RIFV into culture medium from differentiation Day0 to Day3 during adipogenesis. The Oil Red O staining and TG measurement on differentiation Day8 showed that RIFV could suppress the human adipocytes differentiation (Fig. 4a and b).The same result was observed that the Fabp4 mRNA expression level of RIFV-treated adipocytes on differentiation Day8 was obviously lower than that of control adipocytes (Fig. 4c). However, at the late stage, the human adipocyte was treated by RIFV from differentiation Day6 to Day8 during adipogenesis and at D8 the treated group showed no difference with the negative control (Fig. 4d–f).

### C/EBP-β overexpression can rescue the inhibitory effects of RIFV on adipogenesis

**Fig. 5:**
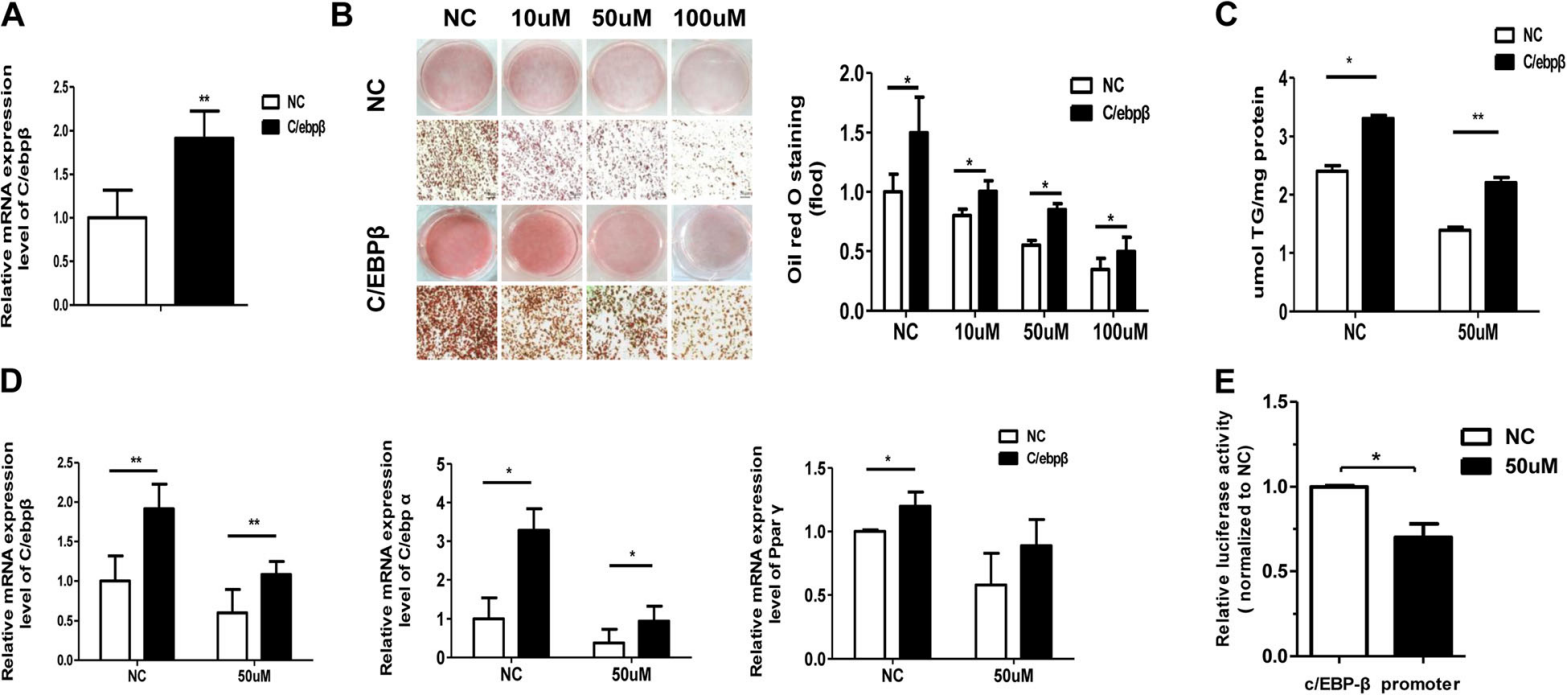
C/EBP-β overexpression could rescue the inhibitory effects of RIFV on human white adipocytes. **a** The C/EBP-β plasmid was transfected into human white preadipocytes for 48 h, and then the expression level of C/EBP-β in preadipocytes overexpressing C/EBP-β was detected by qRT-PCR. **b** Human preadipocytes overexpressing C/EBP-β were treated with different concentrations of RIFV (NC, 10 μM, 50 μM and 100 μM) and cultured to mature adipocytes. The cells were then stained with Oil Red O and visualized under a light microscope (× 200). **c** The cellular triglyceride contents of mature adipocytes stimulated with 50 μM RIFV were measured. **d** The mRNA levels of C/EBP-β, C/EBP-α and PPAR-γ genes in C/EBP-β-overexpressing mature adipocytes treated with RIFV on differentiation day 4. Each value was normalized to PPIA. **e** Luciferase assay using human white adipocytes were transfected with a plasmid containing C/EBP-β-promoter gene and treated with 50 μM RIFV. Luciferase activity was normalized to renilla activity, and the relative luciferase activity was then compared to that of cells with scrambled control peptides. The data are expressed as the means ± SD of three independent experiments. *P < 0.05, **P < 0.01.

To detect whether C/EBP-β played an important role in the inhibitory effect of RIFV on adipogenesis, we transfected C/EBP-β overexpressing plasmids into hWA cells with RIFV treatment on the indicated days. The success of C/EBP-β overexpression is demonstrated in Fig. 5a, as the C/EBP-β expression level was elevated almost 2.0-fold compared with that in the mock cells. Then, we investigated the effect of C/EBP-β overexpression on adipogenesis in RIFV-treated cells by oil red O staining and TG assay on the indicated days. The results in Fig. 5b and c show that the overexpression of C/EBP-β could result in the promotion of differentiation and rescue the inhibitory effect of RIFV on adipocyte differentiation. Furthermore, we found that the overexpression of C/EBP-β in RIFV-treated adipocytes significantly elevated the mRNA levels of C/EBP-α and PPAR-γ when compared to those in negative control cells (Fig. 5d).

To test if RIFV directly regulates C/EBP-β expression on transcription level, we generated a luciferase reporter vector containing the C/EBP-β promoter. Addition of RIFV and transfection of the C/EBP-β luciferase expression plasmid in hWA cells resulted in a significant decrease in luciferase activity (Fig. 5e), suggesting a direct inhibition of RIFV on the C/EBP-β mRNA transcription expression,

## Discussion

We demonstrated that the peptide RIFV is a novel regulator of human adipocyte differentiation. The peptide RIFV was firstly identified from our peptide database, and gain-of-function studies in human and mouse preadipocytes suggested that RIFV could inhibit adipocyte differentiation. In addition, RIFV was investigated to determine its role in the regulation of the adipogenic gene C/EBP-β. These findings indicated that RIFV acts as a negative regulator of adipogenesis in human and mouse preadipocytes.

Obesity is a state of abnormal fat accumulation, and hyperplasia (proliferation) and hypertrophy (differentiation) are the two main mechanisms. Thus, we investigated the functions of RIFV on obesity from the two aspects. In our study, the differentiation of human white adipocytes was remarkably and dose-dependently inhibited by the peptide RIFV, as supported by Oil Red O staining and intracellular TG measurement. Furthermore, the mRNA and protein levels of adipogenesis-associated key regulators C/EBP-β and C/EBP-α were downregulated in adipocytes after treatment with RIFV. We also found that the proliferation and apoptosis of human white adipocytes was not affected when exposed to the peptide RIFV. In addition, a similar inhibition of RIFV on mouse white adipocyte differentiation was revealed. These findings indicate that the suppression of RIFV on adipocyte differentiation is conserved and that RIFV may combat obesity through its negative effect on hypertrophy.

Previous studies have focused on the molecular mechanism of adipogenesis. The C/EBP family and PPAR-γ [23] are important transcriptional factors during adipocyte differentiation. C/EBP-β is induced at the early stage of cell differentiation, which plays a significant role in adipogenesis. C/EBP-α and PPAR-γ are involved in the late stage, which is essential to differentiate preadipocytes into mature adipocytes. These transcription factors act together at both the gene and protein levels. Many studies have confirmed that inhibiting the expression of C/EBP-β is closely related to the weakening of adipocyte differentiation [24]. In this study, RIFV was investigated to determine its role in the regulation of the C/EBP-β gene. Notably, C/EBP-β, and C/EBP-α levels were downregulated in human and mouse adipocytes treated with RIFV. Therefore, understanding how the various molecules regulate adiposity may lead to the development of novel therapeutic approaches to human obesity.

In this study, the peptide RIFV was identified from the functional domains of the precursor protein Titin, which plays an important role in the movement generated by skeletal and cardiac muscle contraction [25]. Titin is mainly expressed in striated muscles and reported to be involved in the formation of cardiac muscle fibers and muscle tissue morphogenesis [26]. In addition, the mutation of Titin could result in familial dilated cardiomyopathy [27, 28]. Previously, our group found that RIFV was abundantly expressed in cardiac cells but not in exogenous cultured adipocytes (data not shown).In this study, we screened that this peptide RIFV could participate in the differentiation process of human and mouse preadipocytes. These results suggested that RIFV may act as a potential endocrine molecule in inter organ cross talk to connect the striated muscles with fat tissues, similar to the action of atrial natriuretic peptide ANP as an activator of the lipolysis of adipose tissues [29] and gastric peptide Nesfatin-1 on the regulation of brown adipocyte differentiation [30]. However, this hypothesis remains to be confirmed by further detection.

## Conclusions

In conclusion, our present study revealed that peptide RIFV plays an important role in human adipocyte differentiation by downregulating the transcriptional factors C/EBP-β. Therefore, RIFV, as a novel peptide, might offer a protective and therapeutic strategy in the management of obesity and obesity-related complications.

## Data Availability

All data generated or analyzed during this study are included in this published article.

## Abbreviation

ATGL: adipose triglyceride lipase
C/EBP-α: CCAAT/enhancer-binding protein alpha
C/EBP-β: CCAAT/enhancer-binding protein beta
CCK-8: WST-8 Cell Counting Kit-8;
FABP4: Fatty acid binding protein 4
GLP-1: glucagon-like peptide 1
PPAR-γ: peroxisome proliferator-activated receptor gamma
TG: Triglyceride
